# Sex-specific developmental plasticity of generalist and specialist predatory mites (Acari: Phytoseiidae) in response to food stress

**DOI:** 10.1111/j.1095-8312.2010.01593.x

**Published:** 2011-03

**Authors:** Andreas Walzer, Peter Schausberger

**Affiliations:** Institute of Plant Protection, Department of Applied Plant Sciences and Plant Biotechnology, University of Natural Resources and Applied Life Sciences1190 Vienna, Austria

**Keywords:** adaptive canalization hypothesis, diet specificity, environmental stress, ephemeral food resource, reaction norm

## Abstract

We studied developmental plasticity under food stress in three female-biased size dimorphic predatory mite species, *Phytoseiulus persimilis*, *Neoseiulus californicus*, and *Amblyseius andersoni*. All three species prey on two-spotted spider mites but differ in the degree of adaptation to this prey. *Phytoseiulus persimilis* is a specialized spider mite predator, *N. californicus* is a generalist with a preference for spider mites, and *A. andersoni* is a broad generalist. Immature predators were offered prey patches of varying density and their survival chances, dispersal tendencies, age and size at maturity measured. *Amblyseius andersoni* dispersed earlier from and had lower survival chances in low density prey patches than *N. californicus* and *P. persimilis*. Age at maturity was not affected by prey density in the generalist *A. andersoni*, whereas both the specialist *P. persimilis* and the generalist *N. californicus* accelerated development at low prey densities. Species-specific plasticity in age at maturity reflects opposite survival strategies when confronted with limited prey: to prematurely leave and search for other food (*A. andersoni*) or to stay and accelerate development (*P. persimilis*, *N. californicus*). In all species, size at maturity was more plastic in females than males, indicating that males incur higher fitness costs from deviations from optimal body size. © 2011 The Linnean Society of London, *Biological Journal of the Linnean Society*, 2011, **102**, 650–660.

## INTRODUCTION

Developmental plasticity in age and size at maturity allows organisms to adaptively match trait expression to environmental gradients (Pigliucci, 2001). The fitness implications of age and size at maturity may differ between males and females, especially in sexually size dimorphic species, possibly resulting in sex-specific reaction norms (Blanckenhorn, 2005). Potential ultimate explanations for species- and sex-specific life-history plasticity can be deduced from the adaptive canalization hypothesis. Traits closely linked to fitness are assumed to be canalized via past selection, which leads to high robustness against environmental stress and low phenotypic plasticity (Schmalhausen, 1949; Stearns & Kawecki, 1994; Nylin & Gotthard, 1998; Stillwell *et al*., 2010). Because the life-history components age, size, and growth depend on each other (Nylin & Gotthard, 1998; Roff, 2001), canalization of one life-history trait is commonly associated with a more plastic response of the other. Thus, knowledge of species- and sex-specific plasticity in age and size at maturity may help to predict the fitness relevance of these key life-history traits.

In most invertebrate species, including arthropods, females are larger than males (Darwin, 1871; Thornhill & Alcock, 1983; Honek, 1993). Although largely ignored in life-history models (Crowley, 2000), empirical studies provide evidence for sex-specific life-history plasticity in female-biased size dimorphic arthropods subjected to environmental stress such as extreme climatic conditions, competition, predation threat or food limitation (Nylin & Gotthard, 1998; Blanckenhorn, 2000, 2005; Teder & Tammaru, 2005; Stillwell *et al*., 2010). Food stress can either arise from limited infinite (i.e. constantly replenished) or limited finite (i.e. diminishing) food availability. In the latter case, selection should favour accelerated juvenile development, which increases the survival probabilities at the expense of smaller body size at maturity (Abrams *et al*., 1996). Such a developmental plasticity pattern was for example found in the desert amphibian *Scaphiopus couchii* (Newman, 1994), the dung fly *Scathophaga stercoraria* (Blanckenhorn, 1999), and the seed beetle *Callosobruchus maculatus* (Møller, Smith & Sibly, 1990), all of which appear to be well-adapted to ephemeral food resources. However, to our knowledge, only one study has dealt with the influence of a limited finite food resource on sex-specific life-history plasticity in arthropods. The male-biased size dimorphic dung fly *S. stercoraria* emerged earlier at smaller size in response to food stress, although the reaction norm was not affected by sex (Blanckenhorn, 1998).

In the present study, we investigated sex-specific phenotypic plasticity of three sexually size dimorphic predatory mite species: *Phytoseiulus persimilis* Athias-Henriot, *Neoseiulus californicus* (McGregor) and *Amblyseius andersoni* (Chant) (Acari: Phytoseiidae), all of which are subjected to limited finite food during juvenile development. All three species are plant-inhabiting predators and co-occur in the Mediterranean region sharing spider mites of the genus *Tetranychus* as prey (De Moraes *et al*., 2004; A. Walzer, pers. observ.). Spider mites are characterized by rapidly succeeding phases of host plant colonization, population growth, dispersal, and local extinction (Sabelis & Dicke, 1985) and thus constitute an ephemeral food resource for their predators. The three phytoseiid species differ in the degree of adaptation to spider mite prey, with *P. persimilis* being highly specialized on spider mites, *N. californicus* being a generalist but having a preference for spider mites, and *A. andersoni* being a broad generalist poorly adapted to utilize spider mites (McMurtry & Croft, 1997).

The juvenile developmentaI phase of phytoseiid mites is rather short relative to their total lifespan (Amano & Chant, 1977; Gotoh, Yamaguchi & Mori, 2004; Walzer & Schausberger, 2008) passing through three mobile stages: larva, protonymph, and deutonymph. Males are polygynous and approximately 20–30% smaller than females (Schulten, 1985; A. Walzer & P. Schausberger, pers. observ.). Males have higher lifetime mating frequencies than females because females need only a single or a few matings to achieve full egg production, whereas males usually inseminate numerous females (up to 50) during life (Schulten *et al*., 1978; Amano & Chant, 1978a; Pappas, Broufas & Koveos, 2005). Female lifetime mating frequencies differ among *P. persimilis*, *N. californicus*, and *A. andersoni*. *Phytoseiulus persimilis* females need a single mating for maximum egg production but re-mate occasionally (Enigl & Schausberger, 2004), whereas *N. californicus* and *A. andersoni* females need multiple matings (Amano & Chant, 1978b; Gotoh & Tsuchiya, 2008). Thus, at similar tertiary sex ratios (Sabelis & Janssen, 1994), the operational sex ratio is more strongly male-biased in *P. persimilis* than *N. californicus* and *A. andersoni*, allowing *P. persimilis* males fewer mating opportunities during life and consequently increasing the relative fitness value of single mating events.

On the basis of species- and sex-specific differences in adaptation to ephemeral spider mite prey and mating behavior of *P. persimilis*, *N. californicus*, and *A. andersoni*, we pursued three major hypotheses. (1) The ability to cope with limited finite spider mite prey by accelerating juvenile development correlates with the degree of adaptation to this prey (Blanckenhorn, 1999). Developmental time plasticity should be linked with dispersal tendencies and survival probabilities at low prey densities. (2) The reaction norm in body size along a food gradient differs between males and females. There is no consistent trend across species in sex-specific body size plasticity when morphological measures such as body length are used as indicators of body size (Stillwell *et al*., 2010) but, for the reasons given below, in the phytoseiid mites investigated in the present study, body size plasticity should be higher in males than females. Large female body size has great advantages in fecundity and mating behavior (A. Walzer & P. Schausberger, unpubl. data) and female lifetime mating frequencies are lower than those of males. Hence, being smaller than standard should be more costly for females than males, resulting in lower female body size plasticity. (3) The degree of male body size plasticity depends on the mating system. Because of fewer mating opportunities, deviation from the optimal body size should be linked with higher fitness costs in males of *P. persimilis* than those of *N. californicus* and *A. andersoni*. Consequently, male body size should be more strongly canalized in *P. persimilis*.

## MATERIAL AND METHODS

### Species origin and rearing

*Phytoseiulus persimilis*, *N. californicus*, and *A. andersoni* used in experiments were derived from laboratory-reared populations founded with specimens collected in Trapani, Sicily, in 2007. Rearing units consisted of plastic tiles resting on water-saturated foam cubes in plastic boxes half-filled with water. The edges of the tiles were covered with moist tissue paper to confine the predators to the rearing arenas. To prevent contamination of the predator populations an adhesive (Raupenleim, Avenarius Agro) was applied on the rim of the plastic boxes and the boxes were placed in a tray containing water with dishwashing detergent. The predators were fed in 2–3-day intervals with *Tetranychus urticae* (Acari: Tetranychidae) by adding bean leaves infested with spider mites (for *P. persimilis*, *N. californicus*) or by brushing spider mites from infested leaves (for *A. andersoni*) onto arenas. Cotton wool tufts under cover slips served as shelter and oviposition sites for *A. andersoni*.

### Experimental units

Each experimental unit consisted of a single detached bean leaf (approximately 4 cm^2^) placed upside down on a water-saturated foam cube in a plastic box half-filled with water. The leaf arena was delimited by strips of moist tissue paper preventing the mites from escaping. To obtain a given prey density, one to four spider mite females were allowed to oviposit on each experimental unit for 24 h. After removing the spider mite females and most of their webbing, the number of spider mite eggs was adjusted to predetermined densities (*N* = 6, 8, 10, 12, 16, 18, 20, 24, 28, 32, 36, 40, 44, and 48 eggs for *A. andersoni*, with 18 to 39 replicates per density, and *N* = 5, 6, 8, 10, 12, 14, 20, 28, and 32 eggs each for *P. persimilis* and *N. californicus*, respectively, with 16–24 replicates per density). Higher prey densities were needed for *A. andersoni* because of its much higher prey demands (Zhang & Croft, 1994).

### Experimental procedures

Eggs aged less than 36 h of *A. andersoni*, *N. californicus*, and *P. persimilis* were singly placed on experimental leaf arenas. The developmental progress of the predatory mites was recorded twice a day in 8- and 16-h intervals until the predators reached adulthood, died on the leaf or escaped from the leaf. Escapers were those individuals that were found dead in the moist tissue surrounding the leaf arena or disappeared completely. After reaching adulthood, each mite was mounted in a drop of Hoyer's medium (Krantz & Walter, 2009) and the microscope slides were dried at room conditions for 2 days. The dorsal shield length of males and females was measured under the microscope at 200× magnification. Dorsal shield length is considered an appropriate indicator of the body size of phytoseiid mites (Croft, Luh & Schausberger, 1999).

### Statistical analysis

All statistical analyses were performed using SPSS for Windows 15.0 (SPSS Inc.). First, the effects of prey density on dispersal (proportion of individuals escaping from the prey patch before reaching adulthood) and survival (proportion of individuals surviving until adulthood within the prey patch) were analyzed by binary logistic regression for each species separately. Species differences in dispersal and survival (data pooled from prey densities 6, 8, 10, 12, 20, 28, and 32 eggs) were analyzed by binary logistic regression using species as categorical covariate. Second, the influence of prey density on age and size at maturity was analyzed with separate linear regressions for each species and sex. Age (average heritability *h*^2^∼0.26) and body size (*h*^2^∼0.4) at maturity are partially genetically determined (Roff, 2001). Thus, we analyzed the influence of prey density on age and size at maturity only at prey densities allowing > 75% predator survival to avoid an inadvertent bias possibly caused by body size and or age-specific survival at low prey densities (individuals with genetically determined small body size and/or rapid juvenile development could survive more likely than larger and/or slower developing individuals). Additionally, multivariate analysis of variance (MANOVA) with subsequent univariate analysis of variance was used to analyze the effects of predator species, prey density, and sex on age and size at maturity of *N. californicus* and *P. persimilis. Amblyseius andersoni* could not be included in this analysis as a result of differing prey density treatments. Third, to detect sex-specific responses to food stress resulting in differential plasticity in age and/or size of males and females, we calculated the sexual size dimorphism index [(female size/male size) − 1] and a sex-specific developmental time index [(female age/male age) − 1]*sensu*Lovich & Gibbons (1992). Size and age indices were normally distributed (Kolmogorow-Smirnov, *P* > 0.05). The influence of prey density on these indices was analyzed with a separate linear regression for each species. Significant *r*^2^ values indicated sex-specific plasticities.

## RESULTS

### Effects of food limitation on species-specific dispersal and survival

**Figure 1:**
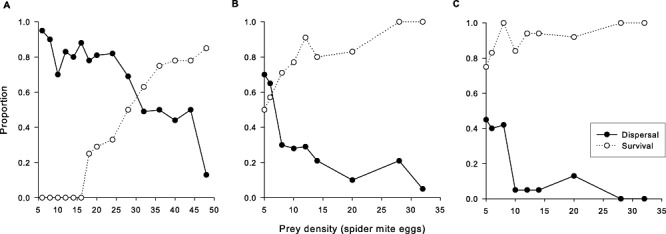
Effect of prey density on dispersal (proportion of individuals leaving the prey patch before reaching adulthood) and survival (proportion of individuals reaching adulthood within the prey patch) of *Amblyseius andersoni* (A), *Neoseiulus californicus* (B), and *Phytoseiulus persimilis* (C).

Prey density influenced the proportion of dispersing individuals and the proportion of individuals surviving until adulthood in all three species (binary logistic regression, d.f. = 1: dispersal: *A. andersoni*: Wald = 47.747, *P* < 0.001; *N. californicus*: Wald = 18.111, *P* < 0.001; *P. persimilis*: Wald = 14.242, *P* < 0.001; survival: *A. andersoni*: Wald = 27.150, *P* < 0.001; *N. californicus*: Wald = 10.250, *P* = 0.001; *P. persimilis*: Wald = 4.649, *P* = 0.031). Most *A. andersoni* juveniles (approximately 80%) prematurely left prey patches containing < 24 spider mite eggs. The percentage of dispersing *A. andersoni* dropped to 50% at prey densities of 32–44 eggs. Only at the highest prey density (48 eggs), 87% *A. andersoni* remained in the prey patch (Fig. 1A). Approximately two thirds of *N. californicus* juveniles (70%) dispersed from the lowest density patches (five or six eggs). The percentage of dispersing *N. californicus* continuously decreased with increasing prey densities, reaching 5% at a density of 32 eggs (Fig. 1B). Half of the *P. persimilis* juveniles dispersed from the lowest density patches (five or six eggs). All juvenile *P. persimilis* remained in the prey patches at densities of 28–32 eggs (Fig. 1C). Species comparisons revealed that significantly more *A. andersoni* juveniles prematurely left the prey patches than juveniles of *P. persimilis* and *N. californicus* (binary logistic regression, d.f. = 1: *A. andersoni* versus *P. persimilis*: Wald = 86.703, *P* < 0.001; *A. andersoni* versus *N. californicus*: Wald = 62.604, *P* < 0.001), whereas more juveniles of *N. californicus* than *P. persimilis* prematurely left the prey patches (Wald = 5.239, *P* = 0.022) (Fig. 1A, B, C). No *A. andersoni* reached adulthood below a prey density of 16 eggs. Even at a prey density of 32 eggs, 50% *A. andersoni* juveniles died (Fig. 1A). Approximately 75% *P. persimilis* and 50% *N. californicus* survived until adulthood when provided with five spider mite eggs (Fig. 1B, C). *Phytoseiulus persimilis* had higher survival chances than both other species, *N. californicus* had higher survival chances than *A. andersoni* (binary logistic regression, d.f. = 1: *A. andersoni* versus *P. persimilis*: Wald = 38.919, *P* < 0.001; *A. andersoni* versus *N. californicus*: Wald = 28.627, *P* < 0.001; *P. persimilis* versus *N. californicus*: Wald = 6.285, *P* = 0.012) (Fig. 1A, B, C).

### Effects of food limitation on age and size at maturity

**Figure 2:**
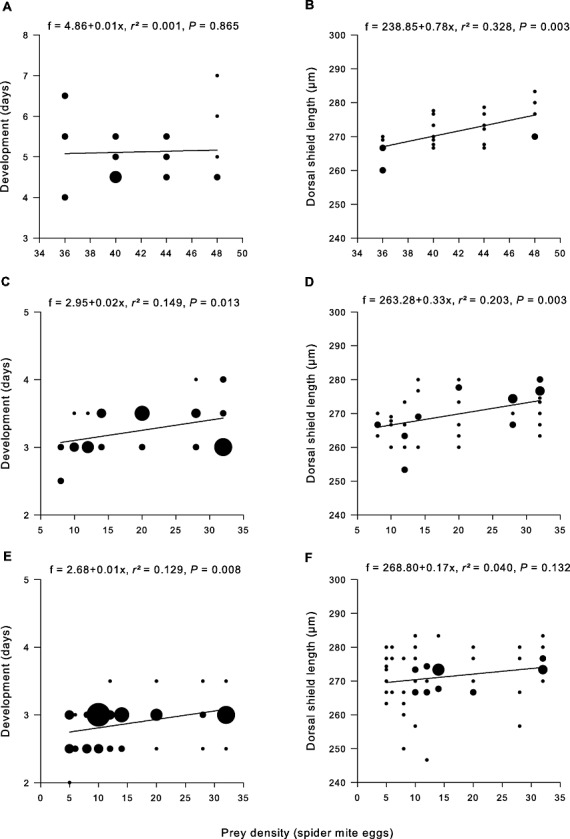
Age and size at maturity of males of *Amblyseius andersoni* (A, B), *Neoseiulus californicus* (C, D), and *Phytoseiulus persimilis* (E, F) regressed on prey densities. Symbol size is proportional to the sample size.

**Figure 3:**
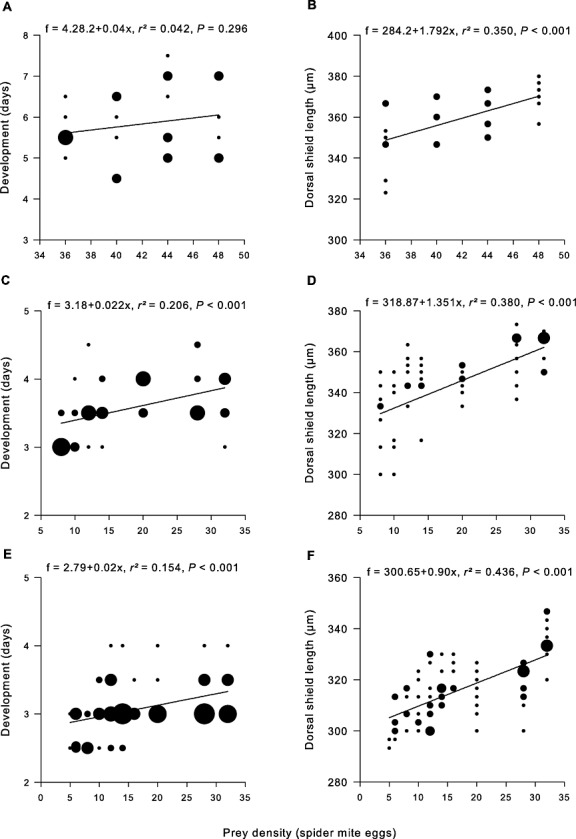
Age and size at maturity of females of *Amblyseius andersoni* (A, B), *Neoseiulus californicus* (C, D), and *Phytoseiulus persimilis* (E, F) regressed on prey densities. Symbol size is proportional to the sample size.

Irrespective of sex, the developmental time of *A. andersoni* was not influenced by prey density (Figs 2A, 3A), whereas body size at maturity was positively correlated with prey density (Figs 2B, 3B). Both male and female *N. californicus* accelerated development (Figs 2C, 3C) and reached adulthood at smaller size with decreasing prey densities (Figs 2D, 3D). Similarly, male and female *P. persimilis* had shorter developmental times at low prey densities (Figs 2E, 3E), which correlated with smaller body size of females (Fig. 3F), but had no influence on male size (Fig. 2F). MANOVA revealed the main effects of predator species, prey density, and sex on age and size at maturity and significant two-way interaction effects on size but not age at maturity (Table 1). *Phytoseiulus persimilis* reached adulthood earlier than *N. californicus* did. Males developed faster than females. Development was shorter at low prey densities (Figs 2C, E, 3C, E). Male *P. persimilis* and male *N. californicus* were similarly sized, whereas *N. californicus* females were larger than *P. persimilis* females (predator × sex interaction). Body size plasticity was higher in *N. californicus* than *P. persimilis* (species × prey interaction) but this was only true for males (prey × sex interaction) (Figs 2D, F, 3D, F).

**Table 1:** The effects of predator (*Neoseiulus californicus, Phytoseiulus persimilis*), sex and prey density on age and size at maturity

Source of variation	Dependent variable	d.f.	*F*	*P*
Predator	Age	1	54.237	< 0.001
	Size	1	26.070	< 0.001
Sex	Age	1	24.681	< 0.001
	Size	1	1505.856	< 0.001
Prey density	Age	8	2.883	0.005
	Size	8	21.193	< 0.001
Predator × Sex	Age	1	2.291	0.132
	Size	1	89.631	< 0.001
Predator × Prey density	Age	8	0.340	0.949
	Size	8	5.827	< 0.001
Prey density × Sex	Age	8	0.532	0.831
	Size	8	8.327	< 0.001

Results of multivariate analysis of variance (MANOVA): Predator: *Pillai trace* = 0.285, d.f. = 2, *P* < 0.001; sex: *Pillai trace* = 0.887, d.f. = 2, *P* < 0.001; prey density: *Pillai trace* = 0.548, d.f. = 16, *P* < 0.001; predator × sex: *Pillai trace* = 0.321, d.f. = 2, *P* < 0.001; predator × prey density: *Pillai trace* = 0.207, d.f. = 16, *P* < 0.001; prey density × sex: *Pillai trace* = 0.276, d.f. = 16, *P* < 0.001; prey density × predator × sex: *Pillai trace* = 0.053, d.f. = 16, *P* = 0.844.

**Figure 4:**
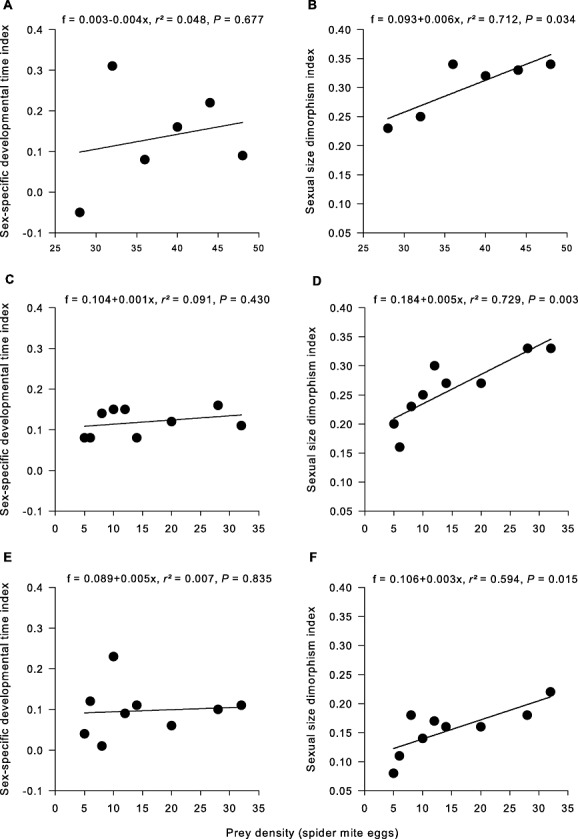
Effect of prey density on the sexual size dimorphism index and the sex-specific developmental time index of *Amblyseius andersoni* (A, B), *Neoseiulus californicus* (C, D), and *Phytoseiulus persimilis* (E, F).

The sex-specific developmental time index was > 0 in all species and at all prey densities (with a single exception at a prey density of 28 eggs in *A. andersoni*), indicating that females needed longer to reach adulthood than males. In no species was the sex-specific developmental time index influenced by prey density. Thus, plasticity in age at maturity did not differ between males and females (Fig. 4A, C, E). By contrast, in all three species, the sexual size dimorphism index decreased with decreasing prey densities, indicating that male body size was more canalized than female body size (Fig. 4B, D, F).

## DISCUSSION

The present study revealed that both adaptation to ephemeral prey and sex are important determinants of developmental plasticity in age and size at maturity of the phytoseiid mites *P. persimilis*, *N. californicus*, and *A. andersoni*. Dispersal tendencies (high for the broad generalist *A. andersoni*, intermediate for the generalist *N. californicus* with a ranked preference for spider mites, low for the spider mite specialist *P. persimilis*) and survival probabilities (low for *A. andersoni*, intermediate for *N. californicus*, high for *P. persimilis*) corresponded to the degree of specialization on spider mite prey of the three species. Better adaptation to spider mite prey was linked with a higher degree of developmental plasticity in age at maturity in both sexes. However, not only the highly specialized *P. persimilis*, but also the generalist *N. californicus* was able to accelerate development at low prey densities. In all three species, the large females were more plastic in size than the small males. Plasticity in male body size was lower in *P. persimilis* than *N. californicus* and *A. andersoni*.

### Species-specific response to food stress

An ephemeral food resource such as a finite prey patch imposes strong selection pressure on juvenile predators to develop efficient survival strategies because limited prey affects the chance to reach adulthood within a given patch. Leaving the prey patch prematurely (before extinction) and searching for more profitable food is one possibility. This appears to comprise a suitable strategy for the highly polyphagous *A. andersoni* because spider mite eggs are a suboptimal food for this predator. We often observed that *A. andersoni* sucked out spider mite eggs only partially, leaving collapsed half-full eggs. The high number of prey eggs needed (including partially consumed eggs) for survival and completion of juvenile development indicates the low profitability of spider mite eggs for *A. andersoni*, probably as a result of poor physiological adaptation and/or inefficient handling. This generalist predator is better adapted to feed on mobile spider mites than eggs (Blackwood, Schausberger & Croft, 2001) and can also utilize other arthropods and non-animal food such as pollen for development and reproduction (McMurtry & Croft, 1997). Phenotypic plasticity of *A. andersoni* in age at maturity was low and mortality was extremely high at low prey densities. Consequently, staying in a patch with limited, finite, and little profitable food should be more risky for *A. andersoni* than to leave the patch and search for alternative food. This assumption is further supported by the dispersal tendencies, which were much stronger in *A. andersoni* than *N. californicus* and *P. persimilis*.

Staying as long as possible in a limited finite spider mite patch and trying to reach adulthood before leaving appears to be a favourable strategy for predators specialized on this type of prey. Premature patch-leaving is much riskier for them than for generalists. Therefore, we assume that specialists have been selected for higher plasticity in developmental time than generalists and accelerate development at the expense of body size under limited finite food availability. Indeed, in both male and female *P. persimilis*, developmental times were positively correlated with spider mite densities. Such a reaction norm may generally evolve in environments where the distribution, quality, and quantity of prey vary unpredictably in space and time and has been observed in mosquitoes, dung flies, and seed beetles (Møller *et al*., 1990; Juliano & Stoffregen, 1994; Blanckenhorn, 1999). Shortening developmental times under limited finite food availability may partly compensate for the costs of reduced body size (Metcalfe & Monaghan, 2001). Faster development could increase the survival probabilities as a result of shortened exposure to con- or heterospecific predators during the vulnerable juvenile period, which would allow earlier dispersal and could finally lead to earlier reproduction and shorter generation times (Stearns, 1992). Interestingly, we observed the same reaction norms in the generalist *N. californicus* and the specialist *P. persimilis*. Similar to *A. andersoni*, *N. californicus* is able to utilize various food sources but, different from *A. andersoni*, *N. californicus* has a clear preference for spider mites among the food types accepted and is well-adapted on this type of prey (McMurtry & Croft, 1997).

### Sex-specific response to food stress

The direction and degree of sex-specific body size plasticity in sexually size dimorphic species varies greatly among species. Recent meta-analyses of insects using body mass as indicator of body size revealed that in most species (60–70%), body size is more plastic in females than males (Teder & Tammaru, 2005; Stillwell *et al*., 2010). However, when morphological traits such as body length were used as indicators, the analysis did no more reveal a general trend in sex-specific size plasticity (Stillwell *et al*., 2010). Additionally, the direction and degree of body size plasticity of males and females not only varies with the type of environmental variable, but also can shift within a given environmental variable from higher to lower female size plasticity (Stillwell *et al*., 2010). Hence, the lack of a general trend suggests that potential explanations for sex-specific size plasticity need to be deduced from species-specific biological and ecological factors.

By contrast to our expectation, in all three predatory mite species, body size was more plastic in females than males. In light of adaptive canalization theory, we assume that deviation from optimal body size is more costly for males than females. It is commonly acknowledged that female and male body size and their plasticities are shaped by differential but inter-related selective forces: fecundity and sexual selection, respectively (Nylin & Gotthard, 1998; Blanckenhorn, 2005), which makes it impossible to pinpoint sex-specific plasticities to single factors. However, overall, it appears that body size plasticity of males is subjected to stronger selective forces than that of females. Small male body size enhances agility, which is advantageous because males not only have to search for prey, but also for receptive females at various spatial scales, such as within and among prey patches within leaves, among leaves within plants, and among plants. Small size is advantageous in these movements because, for example, the mean speed achievable on vertical structures is indirectly proportional to body length (Moya-Laraño, Halaj & Wise, 2002). Additionally, small individuals require less food for maintaining basic body functions allowing them to allocate additional energy to mate search and mating (Andersson, 1994; Blanckenhorn, 2000, 2005). However, being too small may reduce male mating success because disproportional small size is usually disadvantageous in male–male competition and/or female choice (see below for details) (Andersson, 1994).

### Species-specific male body size plasticity under food stress

The reaction norm in male body size was species-specific, with a positive correlation between prey density and male body size in *A. andersoni* and *N. californicus* but not *P. persimilis*. We assume that falling below the optimal body size is linked with higher costs in males of *P. persimilis* than males of *A. andersoni* and *N. californicus*. In general, disproportional small size may lower the mating success of polygynous males for various reasons. Small males are usually inferior in direct competition with larger males (Andersson, 1994; Blanckenhorn, 2000, 2005). Small males approaching females for mating can be rejected (e.g. mites: Enders, 1993; spiders: Schneider & Elgar, 2005) or may be chosen less often than large males if females use size as indicator of male quality (e.g. amphibians: Howard & Young, 1998; reviews: Andersson, 1994; Blanckenhorn, 2000). If females do mate with small males, mating can be disturbed by larger males leading to mating disruption (e.g. mites: Enders, 1993). Moreover, male body size may be positively correlated with the quality and/or quantity of sperm or the size of the ejaculate (e.g. crickets: Wedell, 1997; beetles: Savalli & Fox, 1998). Increased mating frequency could be a strategy to compensate for the disadvantages of a disproportional small body size. This may especially apply to species where females need to mate multiply to maximize their reproductive success. Multiple mating increases total egg production of *N. californicus* and *A. andersoni* (Amano & Chant, 1978b; Gotoh & Tsuchiya, 2008) but does not affect egg output of *P. persimilis* (Schulten *et al*., 1978; Amano & Chant, 1978b). Females should be less willing to re-mate when a single mating is sufficient for maximum egg production than when multiple matings are needed. Consequently, at similar tertiary sex ratios, the operational sex ratio (i.e. the number of receptive females per fertile male) should be higher in species where multiple mating leads to an increase in total egg production such as in *N. californicus* and *A. andersoni*. The costs accruing from missed matings are higher in species where males have less mating opportunities. Therefore, sexual selection should more strongly act on canalization of male body size in species, in which a single mating maximizes total egg production such as in *P. persimilis*.
